# Methyl 3-amino-4-butanamido-5-methyl­benzoate

**DOI:** 10.1107/S1600536808013408

**Published:** 2008-05-14

**Authors:** Xiang Li, Lian-shan Yuan, Dan Wang, Cheng Yao

**Affiliations:** a Department of Applied Chemistry, College of Sciences, Nanjing University of Technolgy, Xinmofan Road No. 5, Nanjing 210009, People’s Republic of China; b Bioengineering Department, Xuzhou Higher Vocational College of Bioengineering, Mine West Road, Xuzhou 221006, People’s Republic of China

## Abstract

The title compound, C_13_H_18_N_2_O_3_, is an inter­mediate in the synthesis of compounds with medicinial applications. The crystal structure is stabilized by inter­molecular N—H⋯O, C—H⋯N and C—H⋯O hydrogen bonds.

## Related literature

For bond-length data, see: Allen *et al.* (1987 ▶). For related literature, see: Engeli *et al.* (2000 ▶); Goossens *et al.* (2003 ▶); Kintscher *et al.* (2004 ▶); Kurtz & Pravenec (2004 ▶); Ries *et al.* (1993 ▶).
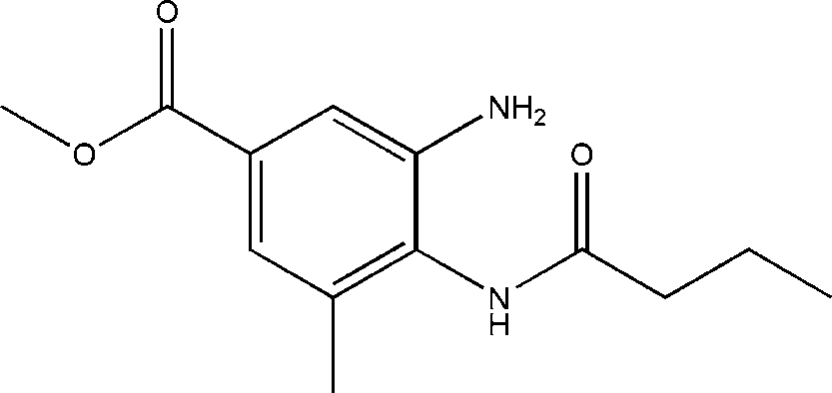


## Experimental

### 

#### Crystal data

C_13_H_18_N_2_O_3_
*M* *_r_* = 250.29
Monoclinic, 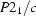

*a* = 10.547 (2) Å
*b* = 16.258 (3) Å
*c* = 8.430 (2) Å
β = 111.69 (3)°
*V* = 1343.2 (5) Å^3^
*Z* = 4
Mo *K*α radiation
μ = 0.09 mm^−1^
*T* = 293 (2) K
0.40 × 0.20 × 0.10 mm

#### Data collection

Enraf–Nonius CAD-4 diffractometer
Absorption correction: ψ scan (North *et al.*, 1968 ▶) *T*_min_ = 0.965, *T*_max_ = 0.991
2579 measured reflections
2404 independent reflections
1511 reflections with *I* > 2σ(*I*)
*R*_int_ = 0.028
3 standard reflections every 200 reflections intensity decay: none

#### Refinement

*R*[*F* ^2^ > 2σ(*F* ^2^)] = 0.074
*wR*(*F* ^2^) = 0.174
*S* = 1.02
2404 reflections
158 parameters
H-atom parameters constrained
Δρ_max_ = 0.50 e Å^−3^
Δρ_min_ = −0.40 e Å^−3^

### 

Data collection: *CAD-4 Software* (Enraf–Nonius, 1989 ▶); cell refinement: *CAD-4 Software*; data reduction: *XCAD4* (Harms & Wocadlo,1995 ▶); program(s) used to solve structure: *SHELXS97* (Sheldrick, 2008 ▶); program(s) used to refine structure: *SHELXL97* (Sheldrick, 2008 ▶); molecular graphics: *SHELXTL* (Sheldrick, 2008 ▶); software used to prepare material for publication: *SHELXL97*.

## Supplementary Material

Crystal structure: contains datablocks global, I. DOI: 10.1107/S1600536808013408/im2061sup1.cif

Structure factors: contains datablocks I. DOI: 10.1107/S1600536808013408/im2061Isup2.hkl

Additional supplementary materials:  crystallographic information; 3D view; checkCIF report

## Figures and Tables

**Table 1:** Hydrogen-bond geometry (Å, °)

*D*—H⋯*A*	*D*—H	H⋯*A*	*D*⋯*A*	*D*—H⋯*A*
N1—H1*A*⋯O1^i^	0.86	2.60	3.141 (4)	122
N2—H2*A*⋯O2^ii^	0.86	2.33	3.077 (4)	145
N2—H2*B*⋯N1	0.86	2.46	2.780 (4)	103
N2—H2*B*⋯O1^i^	0.86	2.36	3.089 (4)	142
C11—H11*A*⋯N1	0.96	2.45	2.901 (5)	108

Symmetry codes: (i) 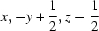; (ii) 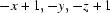.

**Fig. 1.**
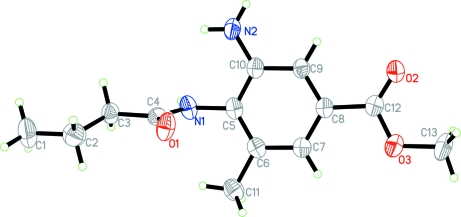
The molecular structure of the title molecule, with the atom-numbering scheme. Displacement ellipsoids are drawn at the 30% probability level.

**Fig. 2.**
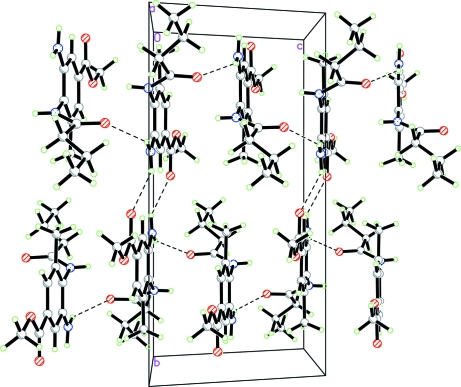
A packing diagram for (I). Hydrogen bonds are shown as dashed lines.

## supplementary crystallographic information

### Comment

3-Amino-4-butyrylamino-5-methyl-benzoic acid methyl ester is important as an intermediate in the synthesis of telmisartan, an angiotensin II receptor blocker, and in the development of obesity and related metabolic disorders in diet-induced obese mice (Ries *et al.*, 1993). Telmisartan can be used as a therapeutic tool for metabolic syndrome, including visceral obesity (Engeli *et al.*, 2000; Kintscher *et al.*, 2004; Goossens *et al.*, 2003; Kurtz *et al.*, 2004). As part of our studies in this area, we report herein the synthesis and crystal structure of the title compound, (I).

In the molecule of (I) (Fig. 1), bond lengths and angles are within normal ranges (Allen *et al.*, 1987). The aromatic ring (C3—C8) is, of course, planar.

The crystal structure is stabilized by intermolecular N—H···O, C—H···N and C—H···O hydrogen bonds (Table 1, Fig. 2).

### Experimental

4-Amino-3-methyl-benzoic acid methyl ester (8.25 g 50 mmol) was acylated with butyryl chloride (5.3 ml 50 mmol) in chlorobenzene at 373 K. The resulting amide was reacted with fuming nitric acid in sulfuric acid (60%) at 273 K. The resulting  4-(butyrylamino)-3-methyl -5-nitrobenzoic acid methyl ester was reduced with hydrogen (5 bar) and palladium (10% on charcoal) in methanol. Then palladium was filtered by suction. The produce separates as a colourless flocculent solid.

Crystals of (I) suitable for X-ray diffraction were obstained by slow evaporation of an ethanolic solution.

### Refinement

H atoms were positioned geometrically, with N—H = 0.86 Å (for NH) and C—H = 0.93, 0.98 and 0.96 Å for aromatic, methene and methyl H, and constrained to ride on their parent atoms, with *U*_iso_(H) = *xU*_eq_(C,*N*), where *x* = 1.5 for methyl H, and *x* = 1.2 for all other H atoms.

### Crystal data

**Table:**

C_13_H_18_N_2_O_3_	*F*_000_ = 536
*M**_r_* = 250.29	*D*_x_ = 1.238 Mg m^−^^3^
Monoclinic, *P*2_1_/*c*	Mo *K*α radiation λ = 0.71073 Å
Hall symbol: -P 2ybc	Cell parameters from 25 reflections
*a* = 10.547 (2) Å	θ = 10–13º
*b* = 16.258 (3) Å	µ = 0.09 mm^−^^1^
*c* = 8.430 (2) Å	*T* = 293 (2) K
β = 111.69 (3)º	Block, colourless
*V* = 1343.2 (5) Å^3^	0.40 × 0.20 × 0.10 mm
*Z* = 4	

### Data collection

**Table:**

Enraf–Nonius CAD-4 diffractometer	*R*_int_ = 0.028
Radiation source: fine-focus sealed tube	θ_max_ = 25.2º
Monochromator: graphite	θ_min_ = 2.1º
*T* = 293(2) K	*h* = −12→11
ω/2θ scans	*k* = 0→19
Absorption correction: ψ scan(North *et al.*, 1968)	*l* = 0→10
*T*_min_ = 0.965, *T*_max_ = 0.991	3 standard reflections
2579 measured reflections	every 200 reflections
2404 independent reflections	intensity decay: none
1511 reflections with *I* > 2σ(*I*)	

### Refinement

**Table:**

Refinement on *F*^2^	Secondary atom site location: difference Fourier map
Least-squares matrix: full	Hydrogen site location: inferred from neighbouring sites
*R*[*F*^2^ > 2σ(*F*^2^)] = 0.075	H-atom parameters constrained
*wR*(*F*^2^) = 0.174	*w* = 1/[σ^2^(*F*_o_^2^) + (0.05*P*)^2^ + 1.5*P*] where *P* = (*F*_o_^2^ + 2*F*_c_^2^)/3
*S* = 1.02	(Δ/σ)_max_ = 0.002
2404 reflections	Δρ_max_ = 0.50 e Å^−^^3^
158 parameters	Δρ_min_ = −0.40 e Å^−^^3^
Primary atom site location: structure-invariant direct methods	Extinction correction: none

### Special details

**Table:**

Geometry. All e.s.d.'s (except the e.s.d. in the dihedral angle between two l.s. planes) are estimated using the full covariance matrix. The cell e.s.d.'s are taken into account individually in the estimation of e.s.d.'s in distances, angles and torsion angles; correlations between e.s.d.'s in cell parameters are only used when they are defined by crystal symmetry. An approximate (isotropic) treatment of cell e.s.d.'s is used for estimating e.s.d.'s involving l.s. planes.
Refinement. Refinement of *F*^2^ against ALL reflections. The weighted *R*-factor *wR* and goodness of fit *S* are based on *F*^2^, conventional *R*-factors *R* are based on *F*, with *F* set to zero for negative *F*^2^. The threshold expression of *F*^2^ > σ(*F*^2^) is used only for calculating *R*-factors(gt) *etc*. and is not relevant to the choice of reflections for refinement. *R*-factors based on *F*^2^ are statistically about twice as large as those based on *F*, and *R*- factors based on ALL data will be even larger.

### Fractional atomic coordinates and isotropic or equivalent isotropic displacement parameters (Å^2^)

**Table:**

	*x*	*y*	*z*	*U*_iso_*/*U*_eq_	
N1	0.7939 (3)	0.28652 (17)	0.4910 (3)	0.0609 (8)	
H1A	0.7889	0.2893	0.3870	0.073*	
O1	0.9151 (2)	0.31431 (18)	0.7622 (3)	0.0722 (8)	
C1	1.2201 (4)	0.4441 (3)	0.6344 (6)	0.1018 (16)	
H1B	1.2826	0.4734	0.7305	0.153*	
H1C	1.1791	0.4818	0.5418	0.153*	
H1D	1.2685	0.4023	0.5994	0.153*	
O2	0.3477 (2)	0.05447 (17)	0.6104 (4)	0.0760 (8)	
N2	0.7806 (3)	0.11598 (19)	0.5029 (4)	0.0669 (8)	
H2A	0.7778	0.0632	0.5085	0.080*	
H2B	0.8469	0.1395	0.4845	0.080*	
C2	1.1113 (4)	0.4052 (3)	0.6834 (5)	0.084	
H2C	1.1555	0.3715	0.7836	0.100*	
H2D	1.0630	0.4487	0.7163	0.100*	
O3	0.2717 (2)	0.17791 (16)	0.6464 (3)	0.0690 (7)	
C3	1.0098 (4)	0.3540 (2)	0.5540 (4)	0.0620 (9)	
H3A	1.0570	0.3098	0.5213	0.074*	
H3B	0.9645	0.3872	0.4533	0.074*	
C4	0.9036 (3)	0.31730 (19)	0.6119 (4)	0.0483 (8)	
C5	0.6835 (3)	0.2489 (2)	0.5237 (4)	0.0522 (8)	
C6	0.5855 (3)	0.2967 (2)	0.5521 (4)	0.0543 (8)	
C7	0.4796 (3)	0.2576 (2)	0.5839 (4)	0.0537 (8)	
H7A	0.4141	0.2888	0.6061	0.064*	
C8	0.4715 (3)	0.1723 (2)	0.5825 (3)	0.0479 (8)	
C9	0.5702 (3)	0.1258 (2)	0.5536 (4)	0.0511 (8)	
H9A	0.5644	0.0687	0.5541	0.061*	
C10	0.6789 (3)	0.1628 (2)	0.5235 (4)	0.0515 (8)	
C11	0.5897 (4)	0.3891 (2)	0.5480 (5)	0.0723 (11)	
H11A	0.6766	0.4066	0.5478	0.108*	
H11B	0.5768	0.4109	0.6468	0.108*	
H11C	0.5185	0.4088	0.4467	0.108*	
C12	0.3588 (3)	0.1281 (2)	0.6125 (4)	0.0540 (8)	
C13	0.1601 (4)	0.1399 (3)	0.6781 (5)	0.0876 (13)	
H13A	0.1050	0.1817	0.7013	0.131*	
H13B	0.1953	0.1038	0.7746	0.131*	
H13C	0.1056	0.1090	0.5794	0.131*	

### Atomic displacement parameters (Å^2^)

**Table:**

	*U*^11^	*U*^22^	*U*^33^	*U*^12^	*U*^13^	*U*^23^
N1	0.0727 (19)	0.078 (2)	0.0357 (14)	−0.0270 (16)	0.0244 (14)	−0.0037 (14)
O1	0.0547 (14)	0.120 (2)	0.0446 (13)	−0.0178 (14)	0.0212 (11)	−0.0041 (13)
C1	0.088 (3)	0.127 (4)	0.099 (3)	−0.049 (3)	0.044 (3)	−0.021 (3)
O2	0.0629 (16)	0.0679 (18)	0.106 (2)	−0.0081 (13)	0.0415 (15)	0.0052 (15)
N2	0.0536 (17)	0.074 (2)	0.082 (2)	−0.0098 (15)	0.0355 (16)	−0.0048 (17)
C2	0.084	0.084	0.084	0.000	0.031	0.000
O3	0.0547 (14)	0.0824 (18)	0.0749 (17)	−0.0004 (13)	0.0296 (13)	0.0048 (13)
C3	0.063 (2)	0.072 (2)	0.059 (2)	−0.0144 (19)	0.0328 (18)	−0.0081 (18)
C4	0.0546 (19)	0.0550 (19)	0.0413 (17)	0.0012 (16)	0.0248 (15)	−0.0036 (15)
C5	0.056 (2)	0.069 (2)	0.0284 (15)	−0.0165 (17)	0.0127 (14)	−0.0038 (15)
C6	0.060 (2)	0.060 (2)	0.0363 (16)	−0.0098 (17)	0.0099 (15)	0.0002 (15)
C7	0.0500 (19)	0.061 (2)	0.0455 (18)	−0.0014 (16)	0.0124 (15)	0.0019 (16)
C8	0.0437 (17)	0.061 (2)	0.0325 (15)	−0.0059 (15)	0.0059 (13)	0.0010 (14)
C9	0.0435 (18)	0.0570 (19)	0.0483 (18)	−0.0042 (15)	0.0118 (15)	0.0038 (15)
C10	0.0456 (18)	0.066 (2)	0.0382 (16)	−0.0081 (16)	0.0102 (14)	−0.0020 (15)
C11	0.082 (3)	0.067 (2)	0.066 (2)	−0.010 (2)	0.025 (2)	0.0051 (19)
C12	0.0473 (19)	0.069 (2)	0.0418 (17)	0.0004 (18)	0.0121 (15)	0.0052 (17)
C13	0.063 (2)	0.123 (4)	0.093 (3)	0.004 (2)	0.048 (2)	0.020 (3)

### Geometric parameters (Å, °)

**Table:**

N1—C4	1.325 (4)	C3—H3A	0.9700
N1—C5	1.430 (4)	C3—H3B	0.9700
N1—H1A	0.8600	C5—C6	1.384 (5)
O1—C4	1.229 (3)	C5—C10	1.400 (5)
C1—C2	1.496 (5)	C6—C7	1.394 (4)
C1—H1B	0.9600	C6—C11	1.503 (5)
C1—H1C	0.9600	C7—C8	1.391 (4)
C1—H1D	0.9600	C7—H7A	0.9300
O2—C12	1.202 (4)	C8—C9	1.379 (4)
N2—C10	1.378 (4)	C8—C12	1.488 (4)
N2—H2A	0.8600	C9—C10	1.399 (4)
N2—H2B	0.8600	C9—H9A	0.9300
C2—C3	1.472 (5)	C11—H11A	0.9600
C2—H2C	0.9700	C11—H11B	0.9600
C2—H2D	0.9700	C11—H11C	0.9600
O3—C12	1.333 (4)	C13—H13A	0.9600
O3—C13	1.439 (4)	C13—H13B	0.9600
C3—C4	1.500 (4)	C13—H13C	0.9600
			
C4—N1—C5	123.7 (2)	C5—C6—C7	118.6 (3)
C4—N1—H1A	118.1	C5—C6—C11	121.8 (3)
C5—N1—H1A	118.1	C7—C6—C11	119.5 (3)
C2—C1—H1B	109.5	C8—C7—C6	120.3 (3)
C2—C1—H1C	109.5	C8—C7—H7A	119.8
H1B—C1—H1C	109.5	C6—C7—H7A	119.8
C2—C1—H1D	109.5	C9—C8—C7	120.0 (3)
H1B—C1—H1D	109.5	C9—C8—C12	117.9 (3)
H1C—C1—H1D	109.5	C7—C8—C12	122.1 (3)
C10—N2—H2A	120.0	C8—C9—C10	121.3 (3)
C10—N2—H2B	120.0	C8—C9—H9A	119.4
H2A—N2—H2B	120.0	C10—C9—H9A	119.4
C3—C2—C1	117.2 (3)	N2—C10—C9	120.9 (3)
C3—C2—H2C	108.0	N2—C10—C5	121.7 (3)
C1—C2—H2C	108.0	C9—C10—C5	117.4 (3)
C3—C2—H2D	108.0	C6—C11—H11A	109.5
C1—C2—H2D	108.0	C6—C11—H11B	109.5
H2C—C2—H2D	107.2	H11A—C11—H11B	109.5
C12—O3—C13	117.1 (3)	C6—C11—H11C	109.5
C2—C3—C4	114.2 (3)	H11A—C11—H11C	109.5
C2—C3—H3A	108.7	H11B—C11—H11C	109.5
C4—C3—H3A	108.7	O2—C12—O3	122.5 (3)
C2—C3—H3B	108.7	O2—C12—C8	123.9 (3)
C4—C3—H3B	108.7	O3—C12—C8	113.6 (3)
H3A—C3—H3B	107.6	O3—C13—H13A	109.5
O1—C4—N1	120.2 (3)	O3—C13—H13B	109.5
O1—C4—C3	123.4 (3)	H13A—C13—H13B	109.5
N1—C4—C3	116.4 (3)	O3—C13—H13C	109.5
C6—C5—C10	122.3 (3)	H13A—C13—H13C	109.5
C6—C5—N1	120.4 (3)	H13B—C13—H13C	109.5
C10—C5—N1	117.2 (3)		
			
C1—C2—C3—C4	−179.7 (4)	C7—C8—C9—C10	−0.7 (4)
C5—N1—C4—O1	0.2 (5)	C12—C8—C9—C10	179.7 (3)
C5—N1—C4—C3	179.6 (3)	C8—C9—C10—N2	176.8 (3)
C2—C3—C4—O1	−15.3 (5)	C8—C9—C10—C5	0.0 (4)
C2—C3—C4—N1	165.4 (3)	C6—C5—C10—N2	−176.9 (3)
C4—N1—C5—C6	79.5 (4)	N1—C5—C10—N2	3.8 (4)
C4—N1—C5—C10	−101.3 (4)	C6—C5—C10—C9	−0.1 (5)
C10—C5—C6—C7	0.9 (5)	N1—C5—C10—C9	−179.3 (2)
N1—C5—C6—C7	−179.9 (3)	C13—O3—C12—O2	−1.1 (5)
C10—C5—C6—C11	−178.5 (3)	C13—O3—C12—C8	−179.6 (3)
N1—C5—C6—C11	0.7 (5)	C9—C8—C12—O2	−1.2 (5)
C5—C6—C7—C8	−1.6 (5)	C7—C8—C12—O2	179.2 (3)
C11—C6—C7—C8	177.8 (3)	C9—C8—C12—O3	177.3 (3)
C6—C7—C8—C9	1.6 (5)	C7—C8—C12—O3	−2.4 (4)
C6—C7—C8—C12	−178.8 (3)		

### Hydrogen-bond geometry (Å, °)

**Table:**

*D*—H···*A*	*D*—H	H···*A*	*D*···*A*	*D*—H···*A*
N1—H1A···O1^i^	0.86	2.60	3.141 (4)	122
N2—H2A···O2^ii^	0.86	2.33	3.077 (4)	145
N2—H2B···N1	0.86	2.46	2.780 (4)	103
N2—H2B···O1^i^	0.86	2.36	3.089 (4)	142
C11—H11A···N1	0.96	2.45	2.901 (5)	108

Symmetry codes: (i) *x*, −*y*+1/2, *z*−1/2; (ii) −*x*+1, −*y*, −*z*+1.

## References

[bb1] Allen, F. H., Kennard, O., Watson, D. G., Brammer, L., Orpen, A. G. & Taylor, R. (1987). *J. Chem. Soc. Perkin Trans. 2*, pp. 1–19.

[bb2] Engeli, S., Negrel, R. & Sharma, A. M. (2000). *Hypertension* **35**, 1270–1277. 10.1161/01.hyp.35.6.1270 10856276

[bb3] Enraf–Nonius (1989). *CAD-4 Software* Enraf–Nonius, Delft, The Netherlands.

[bb4] Goossens, G. H., Blaak, E. E. & Baak, M. A. (2003). *Obes. Rev.* **4**, 43–55. 10.1046/j.1467-789x.2003.00091.x 12608526

[bb5] Harms, K. & Wocadlo, S. (1995). *XCAD4* University of Marburg, Germany.

[bb6] Kintscher, U., Lyon, C. J. & Law, R. E. (2004). *Front. Biosci.* **9**, 359–369. 10.2741/1225 14766373

[bb7] Kurtz, T. W. & Pravenec, M. (2004). *J. Hypertens* **22**, 2253–2261. 10.1097/00004872-200412000-00003 15614015

[bb8] North, A. C. T., Phillips, D. C. & Mathews, F. S. (1968). *Acta Cryst.* A**24**, 351–359.

[bb9] Ries, U. J., Mihm, G. & Narr, B. (1993). *J. Med. Chem.* **36**, 4040–4051. 10.1021/jm00077a007 8258826

[bb10] Sheldrick, G. M. (2008). *Acta Cryst.* A**64**, 112–122. 10.1107/S0108767307043930 18156677

